# Règlementation pharmaceutique au Tchad: état de lieux des pratiques de délivrance des médicaments dans les officines privées de N’Djaména

**DOI:** 10.11604/pamj.2023.44.16.37835

**Published:** 2023-01-09

**Authors:** Levy Ouribe Gueralbaye, Luc Zongo, Ragomzingba Frank Edgard Zongo, Haroun Badawi Mahamat, Arsène Ouédraogo, Djona Atchenemou Avocksouma

**Affiliations:** 1 Université de N'Djaména, Faculté des Sciences de la Santé Humaine, N'Djaména, Tchad,; 2 Université Saint Dominique d'Afrique de l'Ouest, Doulougou, Burkina Faso,; 3 Hôpital Saint Camille, Ouagadougou, Burkina Faso,; 4 Université Nazi Boni, Bobo-Dioulasso, Burkina Faso,; 5 Ministère de la Santé Publique et de la Solidarité Nationale, N'Djaména, Tchad,; 6 STOP-TB Partnership, Global Drug Facility, Geneva, Switzerland

**Keywords:** Pharmacie, règlementation, officine privée, dispensation pharmaceutique, Tchad, Pharmacy, regulation, private pharmacy, pharmaceutical dispensation, Tchad

## Abstract

**Introduction:**

cette étude visait à évaluer les pratiques de délivrances dans les officines pharmaceutiques privées de la ville de N'Djaména. De façon spécifique, (i) décrire les caractéristiques des officines (ii) décrire les modes de délivrance et (iii) vérifier le respect de la réglementation lors de la délivrance sur prescription médicale et sur conseils.

**Méthodes:**

il s'agissait d'une enquête transversale réalisée de juin à décembre 2020. Les données ont été recueillies en deux étapes sous forme d'entretien avec les pharmaciens d'officines et une observation participative des pratiques de délivrances des médicaments dans les officines.

**Résultats:**

au total, 26 officines soit 50% du nombre total des officines de N'Djaména ont été enquêtées. Il ressort de cette enquête, les principaux résultats suivants: Les officines pharmaceutiques privées de la ville de N'Djaména employaient deux catégories de personnels pour la délivrance des médicaments à l'officine: les pharmaciens et un personnel considéré comme des auxiliaires en pharmacie (les techniciens en pharmacie, les infirmiers et les vendeurs ou personnel «sans qualification en santé» car n'ayant pas reçu une formation dans une école de santé reconnue par le ministère de la santé). Très peu d'officines (8%) disposaient d'un espace de confidentialité réservé aux clients et d'ordonnancier. Les trois modes de délivrance ont été observés dans des proportions plus ou moins égales (30 à 40% des cas de dispensation). La délivrance sur demande du patient étant légèrement prédominante (40%) et la majeure partie des médicaments délivrés sur demande du patient et sur conseil du dispensateur appartenait aux différents tableaux de substances vénéneuses (plus de 70%). L'absence remarquée du pharmacien à l'officine justifie que 84% de demandes du patient étaient adressée aux auxiliaires en pharmacie.

**Conclusion:**

cette étude a montré des insuffisances dans l'application de la règlementation pharmaceutique lors de la délivrance des médicaments dans les officines de la ville de N'Djaména. Des facteurs liés à la gouvernance et aux ressources humaines du secteur pharmaceutique ainsi qu'au niveau d'éducation thérapeutique des populations pourraient expliquer ce gap.

## Abstract (en)

**Introduction:**

the purpose of this study was to evaluate drug dispensing practices in private pharmacies in the city of N'Djamena and, specifically, (I) to describe the characteristics of dispensaries, (ii) describe dispensing practices and (iii) assess regulatory compliance when dispensing on the basis of a prescription and an advice.

**Methods:**

we conducted a cross-sectional survey from June to December 2020. Data were collected in two stages, through interviews with pharmacists and participant observation in drug delivery practices in pharmacies.

**Results:**

in total, 26 pharmacies, or 50% of pharmacies in N'Djamena, were surveyed. The main survey findings were: private pharmacies in the city of N'Djamena employed two categories of staff, namely pharmacists and auxiliary staff (pharmacy technicians, nurses and salespeople or staff with “no health qualifications”. These had not been trained in a health school recognised by the Ministry of Health) to dispense medicines. Very few pharmacies (8%) had a customer confidentiality area and a order book. The three modes of delivery were observed in more or less equal proportions (30 to 40% of dispensations). Dispensing at the patient's request was slightly predominant (40%) and the majority of medicines dispensed at the patient's request and based on dispenser's advice belonged to the different tables of toxic substances (over 70%). The noted absence of the pharmacist from the pharmacy justified why 84% of patients' requests were addressed to the pharmacy assistants.

**Conclusion:**

this study shows low compliance of pharmacies with pharmaceutical regulations for proper dispensing of medicines in the city of N'Djamena. Factors related to pharmaceutical sector governance and human resources management as well as therapeutic patient education could explain this gap.

## Introduction

Dans les pays en voie de développement, la non-observance des bonnes pratiques de délivrances sont réelles. Ainsi, une étude faite dans les officines de la ville de Sikasso en 2004 montre que 63% des clients n'avaient pas d'ordonnances lors des achats des médicaments de liste, 12, 22% venaient avec des ordonnances non valables prendre des médicaments comportant les substances vénéneuses [1].

En pratique, nous pouvons constater que les délivrances dans les officines se font souvent sur simple présentation de «bout de papier», d'un ancien conditionnement du produit demandé ou quelques fois d'une ancienne ordonnance et encore sur demande orale du patient. Avec la promulgation par l'Assemblée Nationale du Tchad (ANT), le 30 août 2000, de la Loi N°024/PR/2000 relative à la pharmacie et qui vise l'organisation de la pharmacie humaine et vétérinaire [2], on a assisté à l'ouverture d'un nombre important d'officines et de dépôts pharmaceutiques privés. Cette loi et d'autres textes règlementaires déterminent les conditions d'autorisation pour créer sur toute l'étendue du territoire du Tchad, des établissements pharmaceutiques du secteur public et privé de vente en gros et en détail des médicaments pour répondre aux besoins de santé de la population. Ainsi on assiste à une augmentation du nombre des établissements pharmaceutiques à partir de 2011. Celle-ci est passée de 22 officines privées, 150 dépôts pharmaceutiques et 05 grossistes importateurs et répartiteurs à 52 officines, 381 dépôts pharmaceutiques, 36 grossistes importateurs et répartiteurs en 2020 [3]. Cependant, l'augmentation des établissements pharmaceutiques nécessite une évaluation des pratiques en vue de s'assurer de la qualité de la dispensation pharmaceutique et de préserver et améliorer la santé de la population.

En effet, le médicament dont le premier but est de guérir n'est pas toujours dépourvu de toxicité. Il peut causer de risques graves pour le patient s'il n'est pas utilisé avec précaution. Par conséquent aucun pharmacien ou auxiliaire en pharmacie ne doit encourager tout ce qui concerne «**les achats directs**» ou «**dispensation sur demande du patient**» de médicaments inscrits sur une liste de substances vénéneuses.

Au Tchad, aucune étude sur la pratique de délivrance des médicaments dans les officines n'a été faite. La présente étude a pour objectif d'évaluer les pratiques de délivrance des médicaments dans les officines pharmaceutiques privée de la ville de N'Djaména au regard des exigences règlementaires pharmaceutiques. Plus spécifiquement, de (i) décrire les caractéristiques des officines (ii) décrire les modes de délivrance des médicaments et (iii) vérifier le respect de la réglementation lors de la délivrance sur prescription médicale ou sur conseils.

## Méthodes

**Cadre de l'étude:** la ville de N'Djaména est la capitale l'État du Tchad et la plus grande ville du pays du point de vue démographique. Elle concentre également la plupart des services administratifs et constitue le siège de l'ensemble des représentations diplomatiques. Elle compte à elle seule, 80% des officines du pays. L'étude a eu pour cadre ces officines détenues par des pharmaciens. Les officines privées couvertes par cette étude sont localisées dans 9 communes d'arrondissement sur les 10 que compte la ville de N'Djaména à l'exception du 10^e^ arrondissement.

**Type, période et population de l'étude:** il s'agissait d'une enquête transversale réalisée de juin à décembre 2020. La population cible était constituée des pharmaciens titulaires des officines pharmaceutiques privées; pharmaciens assistants ou remplaçants: auxiliaires en pharmacie dénommés «techniciens supérieurs en pharmacie» au Tchad et clients/patients venant à l'officine avec ou sans ordonnance médicale.

### Critères de sélection

**Étaient inclus dans l'étude:** les pharmaciens d'officine et auxiliaires ayant accepté de participer volontairement à l'étude; tout client venant à l'officine ayant donné son consentement. N'était pas incluse, la population cible non consentante.

**Échantillonnage des officines:** nous avons effectué un échantillonnage stratifié avec comme base d'échantillon la liste des officines privées de la ville de N'Djaména. Cette dernière comptait (au moment de notre étude) 52 officines réparties dans 09 arrondissements, l'enquête a porté sur 26 officines soit 50% du nombre total des officines de N'Djaména (Tableau 1). À partir de la méthode de l'allocation proportionnelle, nous avons déterminé le nombre d'officines à échantillonner dans chaque arrondissement en considérant chaque arrondissement comme une strate. Puis, nous avons procédé à un tirage au sort grâce à un échantillonnage aléatoire simple.

**Tableau 1:** taille de l´échantillon par arrondissement

Arrondissement (strates)	Nombre des officines par Arrondissement	Taille de l´échantillon
1^er^ Arrondissement	01	01
2^e^ Arrondissement	03	01
3^e^ Arrondissement	10	05
4^e^ Arrondissement	06	03
5^e^ Arrondissement	03	01
6^e^ Arrondissement	10	05
7^e^ Arrondissement	11	06
8^e^ Arrondissement	07	03
9^e^ Arrondissement	01	01
10^e^ Arrondissement	00	00
**Total**	**52**	**26**

**Variables de l'étude:** les caractéristiques de l'officine: durée d'existence, existence d'une zone de confidentialité, qualification du personnel chargé de délivrance, la formation des personnels; les modes de délivrance des médicaments observés: sur présentation de l'ordonnance médicale, sur proposition du patient et sur conseil; le statut règlementaire des médicaments délivrés: appartenance à la liste en exploitant les référentiels que sont l'étiquetage sur le conditionnement secondaire et le dictionnaire thérapeutique VIDAL (édition 2020).

**Collecte des données:** à partir des entretiens avec le pharmacien ou l'auxiliaire en pharmacie, de l'observation des séances de dispensation et de l'analyse des prescriptions médicales, les données ont été recueillies à l'aide de deux fiches de collecte: une fiche d'entretien adressée aux pharmaciens titulaires d'officine et/ou à son remplaçant ou assistant et une fiche d'observation active sur les pratiques de délivrance des médicaments. L'observation des pratiques a été faite à raison d'un passage par jour pendant deux jours dans chaque officine aux heures d'ouverture. Pour limiter les fausses déclarations, l'enquêteur sollicitait chaque fois que cela est possible, une preuve de chaque déclaration (par exemple, certificat/attestation et/ou diplôme de formation). Aussi, l'observation des séances de dispensation a été faite dans la discrétion pour ne pas influencer le patient et le dispensateur. Aussi, l'enquêteur était intégré dans l'équipe de dispensation comme un personnel de l'officine durant cette phase d'observation.

**Traitement des données:** les données ont été saisies et analysées à l'aide du logiciel SPSS version 16. Les moyennes et proportions ont été déterminées pour les différentes variables de l'étude: durée moyenne d'existence des officines, proportion des différentes catégories de personnel; proportion d'officines ayant un espace de confidentialité, proportion d'officines disposant d'un ordonnancier, proportion d'officine disposant d'un pharmacien au moment du passage pour les observations des pratiques, proportion des différents modes de délivrance, proportion de médicaments appartenant aux listes de substances vénéneuses.

**Considérations éthiques:** une autorisation de recherche a été délivrée par l'autorité décanale de la Faculté des Sciences de la Santé Humaine (FSSH) de N'Djaména. Une seconde autorisation a été délivrée par la Direction Générale de la Pharmacie du Médicament et de Laboratoire (DGPML). Une troisième autorisation a été obtenue des responsables des officines enquêtées. Par ailleurs, les données ont été recueillies dans l'anonymat, dans la confidentialité, avec le consentement éclairé des participants et dans le respect des différents acteurs intervenant dans cette étude. Les participants (pharmaciens, auxiliaires en pharmacie et patients/clients) non consentants n'ont pas été inclus dans l'enquête.

## Résultats

**Caractéristiques des officines:** la ville de N'Djaména comptait au début de notre étude en juin 2020 officiellement 52 officines, l'enquête a porté sur 26 officines soit 50% du nombre total des officines de N'Djaména.

**Figure 1:**
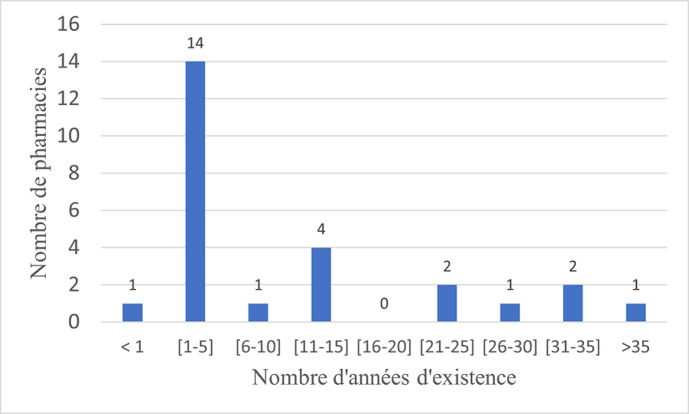
répartition des officines selon le nombre d'années d'existence

***Localisation géographique des officines et durée d'existence:*** parmi les officines enquêtées, 6 (22%) étaient localisées dans le septième arrondissement. La Figure 1 indique la répartition des officines selon le nombre d'années d'existence des officines enquêtées. La durée d'existence moyenne des officines enquêtées était de 5 ans. Les extrêmes étaient de moins d'un an (n=1) une officine) et plus de 35 ans (n=1).

***Existence de zone de confidentialité:*** dans notre échantillon d'étude, deux (02) officines soit 8% avaient une zone de confidentialité bien distincte. Ces officines disposaient de comptoirs éclatés ou d'espace aménagé. Le reste des officines n'en disposait pas. Les patients étaient reçus au comptoir sans prendre de précautions pour garantir leur confidentialité.

***Qualification et effectifs du personnel chargé de la délivrance des médicaments:*** quatre (4) catégories de personnels permanents étaient impliquées dans la délivrance des médicaments: les pharmaciens titulaires ou assistants. Les techniciens supérieurs en pharmacie, les infirmiers et un personnel «sans qualification en santé» qualifiés de «vendeurs» qui jouaient le rôle d'auxiliaires en pharmacie. Les vendeurs d'après la législation ont bénéficié d'un stage à l'officine d'au moins 6 mois et ont un niveau d'études minimal «Brevet d'études du premier cycle ou BEPC». Dans notre étude, 21 (51%) des vendeurs de médicaments avaient le niveau licence. Les autres avaient un niveau d'études inférieur: BEPC et baccalauréat.

Parmi les officines enquêtées, 24 soit 92% étaient tenues par des pharmaciens titulaires de l'officine. Les 2 autres étaient tenues par un pharmacien gérant et un pharmacien remplaçant. Les officines enquêtées employaient au total quatre-vingt-sept (87) auxiliaires. La moyenne des auxiliaires par officine était de trois (03). L'étude a montré que tous les auxiliaires participaient à la délivrance des médicaments. Les auxiliaires étaient répartis comme suit: cinq (05) techniciens supérieurs en pharmacie, soit 6%; quarante un (41) infirmiers, soit 47%; quarante un (41) personnel «sans qualification en santé» ou vendeurs, soit 47%. Le personnel non permanent était constitué d'étudiants stagiaires en pharmacie au nombre de 8 durant la période de l'enquête.

***Formation du personnel:*** dans notre échantillon, huit (08) pharmaciens affirmaient que leurs personnels recevaient des formations continues soit 31%, tandis que 18 (69%) d'entre eux répondaient ne pas assurer une formation continue pour leurs personnels. Par ailleurs, ils affirmaient donner souvent des informations sur la pratique officinale notamment la délivrance des médicaments. La formation continue était dispensée par les pharmaciens et certaines structures privées. Les thèmes de formation cités par les pharmaciens qui affirmaient assurer la formation continue de leurs personnels sont: la dispensation, marketing, gestion de stock assisté par ordinateur; le logiciel Sage commercial et comptabilité; le marketing commercial, l'accueil des patients à l'officine; la prise en charge de trouble digestif, le paludisme, la toux, le cari dentaire; la préparation de la solution hydroalcoolique; le mouillage de l'alcool; la technique de dispensation des médicaments. La prise en charge des pathologies digestives à l'officine (diarrhées, constipation, douleurs abdominales, ballonnements); la prise en charge des pathologies respiratoires à l'officine (rhume, toux…); les conseils à l'officine sur les pathologies courantes (paludisme, …); les conseils à l'officine sur les pathologies chroniques (hypertension artérielle, diabète, asthme).

***Outils de gestion de la dispensation des médicaments:*** les documents utilisés pour la délivrance des médicaments à l'officine étaient: le Dictionnaire thérapeutique Vidal, Edition Afrique francophone: 2010, 2017, 2018, 2019 et 2020; le Guide thérapeutique: Dorosz 2010, 2017, 2018, 2019, 2020; le Petit Larousse de la médecine, édition 2009; Charpentier B, F. Hamon, Lerleac H, L. Ridoux. Guide du préparateur en pharmacie, Masson, 1999; le dictionnaire infirmier, édition Maloine 2007.

**Modes de délivrance des médicaments dans les officines:** trois principaux modes de délivrance des médicaments ont été observés dans les 26 officines enquêtées: la délivrance sur présentation d'une prescription médicale; la délivrance sur conseil du pharmacien et/ou des auxiliaires en pharmacie; la délivrance sur demande du patient. La délivrance sur demande du patient concernait les délivrances soit sur demande orale du patient soit sur présentation d'un ancien emballage ou plaquette ou plaquette du médicament, d'un bout de papier sur lequel est inscrit le nom du produit. Enfin, le téléphone est aussi utilisé pour la proposition des médicaments. Nous avons assisté à deux cent soixante-deux (262) cas de délivrance des médicaments dans les 26 officines reparties dans les différents arrondissements de la ville de N'Djaména. Parmi les délivrances, nous avons observé 81 (31%) cas de délivrance sur prescription médicale, 106 (40%) cas de délivrance sur demande du patient, 75 (29%) cas de délivrance sur conseil. Le nombre total de médicaments délivrés était de mille cent vingt (1120): 371 (33%) de médicaments sur ordonnance médicale, 422 (38%) sur demande du patient, 327 (29%) sur conseil.

***Délivrance sur ordonnance médicale:*** sur l'ensemble des ordonnances médicales exécutées (n=78), 37% étaient conformes sur le plan de la qualité règlementaire. Le nombre total de médicaments prescrit sur ces ordonnances était de 371. Nous avons noté 107 (28.8%) médicaments hors liste, 136 (36.7%) médicaments de liste 1, 126 (34,00%) médicaments de liste 2 et deux (0.5%) de la liste des stupéfiants.

***Qualification des prescripteurs:*** les infirmiers représentaient 44% des prescripteurs et les médecins 26%. Nous avons observé 4% d'ordonnances délivrées par des prescripteurs non qualifié (techniciens de laboratoire) et 7% des ordonnances ne comportaient pas de renseignements sur les prescripteurs.

***Écart entre la date d'exécution et la date de prescription:*** nous avons observé que 32% des ordonnances sont exécutées le même jour de la prescription, 23% sont exécutées sur une durée allant d'un à deux jours, 15% sont exécutées sur une période allant de 3 à 7 jours et 11% sont exécutées sur une période dépassant 7 jours. Il est à noter que sur les 81 ordonnances, 19% ne contiennent aucune date mentionnée.

***Mention des quantités à délivrer:*** sur les 81 prescriptions médicales, 42 (52%) ne mentionnaient pas la quantité de médicaments suffisant pour le traitement.

***Qualification de l'exécuteur de l'ordonnance médicale:*** sur les 81 ordonnances, 5% des ordonnances étaient exécutées par les pharmaciens, 8% par les préparateurs en pharmacie, 43% par des infirmiers, 6% par des étudiants stagiaires en pharmacie, 38% par un personnel sans qualification en santé.

***Attitude face à une ordonnance médicale non conforme:*** l'attitude des pharmaciens face à une ordonnance non conforme était la suivante: rejet de l'ordonnance médicale suivie d'une explication des risques liés à l'exécution de la prescription: 5 pharmaciens (19%); servir les médicaments accompagnés de conseil de bon usage: 12 pharmaciens (46%); référer le patient au prescripteur pour la correction: 6 pharmaciens (23%); contacter le prescripteur pour la reprise de l'ordonnance: 3 pharmaciens (12%).

***Motif du rejet de la prescription médicale:*** les motifs de rejet de la prescription médicale étaient essentiellement: l'illisibilité de l'ordonnance (61.5%); l'absence de cachet sur l'ordonnance pour les psychotropes (11.5%) et la mauvaise orthographe désignant les médicaments (27%).

***Existence d'un ordonnancier:*** aucune officine enquêtée ne disposait de l'ordonnancier. Nous avons observé 5 officines soit 19% qui détenaient les registres des stupéfiants.

***Exécution de l'ordonnance médicale suivie de conseils et/ou explication du traitement au patient:*** sur l'ensemble des officines enquêtées, il y a eu 32 (41%) cas de conseil observés dont 18 (56%) était à la demande du patient et 14 (44%) étaient donnés systématiquement sur les 78 ordonnances exécutées; 46 (59%) exécutions de l'ordonnance n'étaient pas suivi se conseil.

***Description du type de conseils:*** les conseils donnés par le personnel consistaient à essentiellement à retranscrire la posologie mentionnée par les prescripteurs sur la boîte (mode d'emploi, la posologie, les mesures hygiéno-diététiques).

**Délivrance sur conseil du dispensateur:** le nombre de médicaments délivrés sur conseil était de 327. Le nombre de médicaments par proposition variait entre 3 et 4 médicaments. Parmi les médicaments, nous avons noté 177 (54%) médicaments de liste I. Quatre-vingt-onze (28%) médicaments de liste II et 59 (18%) médicaments hors liste. Les classes thérapeutiques conseillées étaient dominées par: les antipaludiques, les antalgiques, les antipyrétiques, les anti-inflammatoires, les antihistaminiques, les antitussifs, les antiparasitaires, les vitamines, les antibiotiques.

***Qualification du personnel ayant conseillé les médicaments:*** au cours de notre étude, nous avons observé que les médicaments étaient conseillés par: le personnel sans «qualification en santé» ou vendeurs: 32%; les pharmaciens: 5%; les étudiants stagiaires en pharmacie: 7%; les techniciens en pharmacie: 11%; les infirmiers: 45%.

***Acceptation du conseil par le patient:*** tous les conseils donnés aux patients à notre présence ont été acceptés et les médicaments servis, suivis de conseils sur le mode d'emploi, la posologie et la durée du traitement.

**Délivrance sur demande du patient (automédication):** sur les 106 demandes de patients, six n'ont pas donné lieu à une dispensation à cause de rupture du médicament demandé. Le nombre total de médicaments délivrés sur proposition des patients est de quatre cent vingt-deux (422) médicaments. Le nombre de médicaments par proposition variait entre 3 à 4.

***Inscription sur les listes des substances vénéneuses:*** parmi les médicaments proposés par les patients, nous avons noté 92 (22%) médicaments hors liste, 111(26%) médicaments de la liste I et 219 (52%) médicaments de la liste II.

***Qualification du personnel ayant reçu la demande du patient:*** nous avons observé que 50% de cas de demande de médicaments étaient adressés aux infirmiers, 29% au personnel sans qualification en santé, 5% aux techniciens en pharmacie présents au comptoir soit total 84% de demandes adressées aux auxiliaires. Le reste des demandes était reçu par les pharmaciens (8%) et par les stagiaires en pharmacie (8%).

***Nombre de médicaments délivrés sur demande du patient:*** sur les cent six (106) cas médicaments demandés, aucun refus n'a été opposé au client sauf pour les cas où une rupture de médicaments a été constée.

**Opinions des pharmaciens sur la vente libre de certains médicaments à l'officine:** au cours de l'étude 12 (46%) pharmaciens estimaient que la vente libre des médicaments constituait une exposition des patients aux risques d'un usage irrationnel des médicaments. Par contre, 10 pharmaciens (39%) avaient un avis favorable quant à cette pratique pour des médicaments dit «conseils» ou de «médication officinale; 4 (15%) avaient un avis favorable pour libéraliser la vente de tous les produits si le ministère n'arrive pas à règlementer le secteur pharmaceutique.

**Consultations de documents sur les médicaments délivrés:** nous avons constaté que le personnel dans les officines consultait rarement les documents mis à leur disposition pour la délivrance des médicaments. Sur 81 ordonnances médicales reçues, seules sept (07) soit 9% ont fait l'objet d'une consultation de documents (dictionnaire Vidal 2018, le Dorosz 2020, internet). De même, quant aux 106 cas de proposition du patient, le personnel ne s'est pas référé aux référentiels thérapeutiques disponibles. Tous les conseils de médicaments aux patients ont été faits sans consultation de référentiels.

## Discussion

Pour la collecte des données, nous avons réalisé des entretiens avec les pharmaciens d'officine pour avoir leur perception ainsi que leur attitude face à la délivrance des médicaments. Nous nous sommes limités à l'aspect règlementaire de délivrance des médicaments. Les informations obtenues auprès des pharmaciens d'officine ont pu être entachées de subjectivités ou d'imprécisions. Nous avons toutefois inclus dans notre méthodologie une observation participative. Par ailleurs, les résultats ne peuvent pas être généralisés à l'échelle du pays parce que les officines enquêtées étaient situées en zone urbaine. Des difficultés ont par ailleurs été rencontrées au cours de cette étude (i) l'absence, souvent du pharmacien titulaire et même d'un assistant à l'officine laissant seuls les auxiliaires qui ne pouvaient pas autoriser l'enquête sans avis du premier responsable mais aussi de répondre à certaines questions; (ii) L'incompréhension de certains auxiliaires qui ne voulaient pas que l'enquêteur s'entretienne avec le patient/client venant sans ordonnance chercher de médicaments.

**Caractéristiques des officines:** la concentration des officines dans un même arrondissement (21% dans l'arrondissement 7) pose le problème d'une répartition inégale des officines dans les arrondissements de la ville de N'Djaména. Nous comptons qu'une seule officine dans le 1^er^ et le 9^e^ arrondissement. Le 10^e^ arrondissement ne dispose d'aucune officine privée. L'étude a révélé que seulement 8% des officines disposaient d'une zone de confidentialité aménagée dans l'espace réservé aux clients pour les recevoir dans la discrétion. Selon l'article 17 du Décret N° 1582/PR/PM/MSP/2015 fixant les conditions d'ouverture et de fonctionnement d'une officine de pharmacie stipule que la superficie, l'aménagement, l'agencement et l'équipement des locaux d'une officine de pharmacie doivent être adaptés à ses activités et permettre le stockage des médicaments et produits pharmaceutiques dans des conditions de bonnes pratiques pharmaceutiques [4]. Ces dispositions sont faiblement respectées. Par ailleurs, l'étude a montré que 92% des officines étaient tenues par des pharmaciens titulaires, 4% par des pharmaciens remplaçants et 4% par des pharmaciens gérants.

Cependant, en pratique, les pharmaciens étaient absents à l'officine. Seulement 15% étaient présents dans leur officine lors de notre passage pour la collecte des données. Ceci pourrait s'expliquer par le fait que ces pharmaciens assument plusieurs responsabilités aussi bien dans le secteur privé que dans le secteur public. Or, l'article 59 de la Loi N°24/PR/2000 du 24 novembre 2000 relative à la pharmacie, stipule que les fonctions de pharmacien responsable sont incompatibles avec toute autre activité engageant le même diplôme. Toutefois, par dérogation à cet article, tout pharmacien peut enseigner à titre vacataire [2]. Aussi les pharmaciens titulaires d'une officine sont autorisés par la loi à se faire assister par un ou plusieurs pharmaciens dits «pharmacien assistant». Cependant nous n'avons pas rencontré un pharmacien assistant au cours de notre étude.

Concernant les auxiliaires en pharmacie, les textes en vigueur au Tchad ne donnent aucune précision sur la définition, les responsabilités, les attributions et le niveau d'instruction des auxiliaires en pharmacie. Cependant, le Décret N°1437/PR/PM/MSP/2015 [5] fixant les conditions d'ouverture et de fonctionnement des dépôts pharmaceutiques à son article 4, autorise les assistants en pharmacie, les préparateurs en pharmacie, les infirmiers diplômés d'État à ouvrir de dépôt pharmaceutique pour la détention et la vente des médicaments et produits pharmaceutiques dont la liste est fixée par arrêté du ministre de la santé publique. Ce même Décret à son article 14 qualifie de vendeur dans les dépôts pharmaceutiques que les personnes au moins titulaires du BEPC ou d'un diplôme équivalent et nantis d'un certificat attestant d'un stage d'une durée d'au moins six mois dans un établissement pharmaceutique agréé par le Ministère de la Santé publique et de la Solidarité nationale.

Le Décret N° 1582/PR/PM/MSP/2015 fixant les conditions d'ouverture et de fonctionnement d'une officine de pharmacie à son article 12 indique qu'en toutes circonstances les médicaments magistraux ou officinaux doivent être préparés par un pharmacien ou sous surveillance directe d'un pharmacien. Ces différents textes, sans préciser la qualification du personnel, autorisent le pharmacien à se faire aider dans ces différents actes officinaux. Notre étude a montré que les officines disposaient des auxiliaires/vendeurs et tous sont affectés à la délivrance des médicaments. Globalement, dans les pays en développement, la situation des ressources humaines à l'officine contraste avec celle des pays développés comme le Bhutan où près de 94% du personnel exerçant à l'officine ont la qualification requise [6].

**Les modes de délivrance des médicaments à l'officine:** nous avons observé 3 principaux modes de délivrance des médicaments: délivrances sur prescription médicale; délivrances sur conseils et délivrances sur demande du patient. Nous avons assisté à 262 cas de délivrances des médicaments au cours de notre enquête dont 31% sur ordonnance médicale, 40% sur proposition du patient et 29% sur conseils. Une étude à Bengalore (en Inde), trouvait un taux de de délivrance sur prescription d'environ 55% [7].

### Délivrance sur ordonnance médicale

**Adéquation de l'ordonnance sur le plan règlementaire:** il s'agissait pour nous d'observer si les ordonnances médicales comportant au moins un médicament de liste respectaient l'aspect réglementaire. L'identification du prescripteur: nom, prénom, adresse complète ainsi que la qualification du prescripteur. La date de prescription. L'identification du patient: nom, prénom, âge et poids du patient. Le ou les médicaments: noms en entier, formes, dosage, durée du traitement. L'authenticité du prescripteur: signature et cachet. Sur cette base, 37% des prescriptions respectaient l'aspect réglementaire. Nos résultats sont inférieurs à ceux de Traoré [8] au Burkina Faso en 2013 qui trouvait 45% et supérieurs à ceux de Ouédraogo en 2010 [9] au Burkina Faso qui trouvait 4,44%.

**Adéquation de l'exécution de la délivrance sur ordonnance:** l'étude a montré que 5% des ordonnances médicales étaient exécutées par les pharmaciens contre 95% exécutées par des auxiliaires. Nos résultats sont comparables à ceux trouvés par Ouédraogo [9] à Ouagadougou en 2010: 7,56% et 90,22% respectivement par les pharmaciens et les auxiliaires. Traoré [8] en 2013 au Burkina-Faso quant à lui rapportait aussi que les ordonnances étaient plus exécutées par les auxiliaires que par les pharmaciens respectivement 86% et 9%. Le problème de l'absence des pharmaciens au comptoir demeure toujours. Une application stricte s'impose quant à la règlementation sur la présence d'un pharmacien au comptoir, afin de permettre une application des règles de délivrance des médicaments dans les officines. Par ailleurs, nous avons observé que 52% des ordonnances reçues ne comportaient pas la quantité de médicaments suffisant pour le traitement mais servi en raison d'une boîte par médicament prescrit. Ceci pourrait favoriser la résistance bactérienne pour les cas des antibiotiques.

**Explication et/ou conseils au patient après exécution de l'ordonnance médicale:** notre étude a montré que les conseils aux patients n'étaient pas automatiquement donnés après la délivrance des médicaments. L'explication des ordonnances se faisait à 56% à la demande du patient. Elle était généralement donnée par tout personnel exerçant à l'officine. Ce constat s'avère inquiétant car une bonne délivrance doit être suivi de conseils d'usage, mais la plupart des délivrances sont exécutées par des vendeurs et les conseils donnés consistaient seulement à reprendre en traçant sur la boîte la posologie mentionnée par les prescripteurs. Ce rôle de conseiller sanitaire devrait être du ressort du pharmacien qui a reçu une formation adéquate en la matière.

**Délivrance sur conseils:** les résultats ont montré que sur 327 médicaments conseillés, 268 (82%) contenaient des substances vénéneuses. Ces résultats sont supérieurs à ceux présentés par Traoré [8] en 2013 au Burkina Faso qui trouvaient 59%. Les vendeurs se basent sur les signes des pathologies pour conseiller les médicaments. Trois cent vingt-sept (327) médicaments délivrés sur 75 conseils donné ce qui revient à délivrer 3 à 4 médicaments à chaque client venant expliquer son problème. L'acte de délivrance des médicaments sur conseils doit toujours s'appuyer sur des connaissances bien établies en sémiologie en physiopathologie et en pharmacologie [10]. Il est impératif que les pharmaciens d'officine exercent leur art dont ils détiennent le monopole.

**Délivrance sur demande du patient:** les résultats ont montré que sur 106 cas de demandes du patient, 422 médicaments ont été délivrés dont 330 médicaments de liste soit 78%; 84% de demandes du patient étaient adressées aux auxiliaires en pharmacie. La délivrance sur demande du patient des médicaments de liste est préjudiciable à l'ensemble du système sanitaire car des études ont montré que plus de la moitié (63%) des ordonnances prescrites à N'Djaména n'étaient pas conformes. Ce qui rend difficile l'application des règles de délivrance des médicaments à l'officine. En Tanzanie, une étude en milieu urbain montrait que la majorité des dispensateurs avait un niveau de connaissance jugé faible à moyen [11].

**La tenue de l'ordonnancier à l'officine:** notre étude a montré qu'aucune officine ne disposait de l'ordonnancier. Ce document réglementaire obligatoire pour le pharmacien d'officine est utilisé pour la transcription des ordonnances des préparations magistrales et de substances vénéneuses délivrées en nature ou sous formes spécialisées. En effet, le Décret N°1582/PR/PM/MSP/2015 fixant les conditions d'ouverture et de fonctionnement d'une officine de pharmacie à son article 12 stipule que toute délivrance par un pharmacien d'une prescription médicale comportant des psychotropes ou stupéfiants doit faire l'objet d'une transcription dans un ordonnancier ou d'un enregistrement par tout autre système approprié portant: le nom et l'adresse de l'officine; un numéro d'ordre différent et chronologique; la date de délivrance de la prescription et le nom et l'adresse du patient. Malgré cela, nous avons observé que 19% des officines enquêtées détenaient les registres des stupéfiants et font rarement des enregistrements. Traoré [8] au Burkina Faso en 2013 trouvait que 12% des officines enregistraient quelques substances vénéneuses dont les plus importants étaient des psychotropes. Cette situation a été constatée par Diarra en 2003 [12] à Bamako avec un taux de respect de 0% du remplissage de l'ordonnancier. Ce faible taux d'enregistrement des médicaments de liste pourrait s'expliquer par: l'absence de rigueur dans l'application de la législation sur ces produits; le fait que les ordonnances médicales n'étaient pas toujours conformes aux exigences règlementaires [13]; le fait que les officines ne disposaient même pas de ce document.

## Conclusion

La délivrance des médicaments est un acte pharmaceutique très important qui exige du pharmacien un respect de la déontologie et de l'éthique professionnelle. Le médicament, présenté comme possédant des propriétés curatives et préventives à l'égard des maladies humaines et animales peut également engendrer des risques graves s'il n'a pas fait l'objet d'un usage rationnel. L'étude s'est déroulée auprès de 26 officines pharmaceutiques privées de la ville de N'Djaména en deux étapes sous forme d'entretien avec les pharmaciens d'officines et une observation participative des pratiques de délivrances des médicaments dans les officines. Au regard de la proportion d'officines enquêtées et la méthode aléatoire utilisée pour la sélection des officines, les résultats peuvent être extrapolés à l'ensemble de la ville de N'Djaména et des autres villes des pays en voie développement dont les caractéristiques socio-démographiques et économiques sont similaires à celles de N'Djaména. Aussi, il ressort de cette enquête, les conclusions suivantes: les officines pharmaceutiques privées de la ville de N'Djaména employaient 2 catégories de personnels pour la délivrance des médicaments à l'officine: Les pharmaciens et un personnel considéré comme des auxiliaires en pharmacie (les techniciens en pharmacie, les infirmiers et les vendeurs ou personnel «sans qualification en santé» car n'ayant pas reçu une formation dans une école de santé reconnue par le ministère de la santé); très peu d'officines disposaient d'un espace de confidentialité réservé aux clients et d'ordonnancier; les trois modes de délivrance ont été observés dans des proportions plus ou moins égales; la délivrance sur demande du patient étant légèrement prédominante et la majeure partie des médicaments délivrés sur demande du patient et sur conseil du dispensateur appartenait aux différents tableaux de substances vénéneuses et l'absence du pharmacien à l'officine justifie que 84% de demandes du patient étaient adressées aux auxiliaires en pharmacie.

### Etat des connaissances sur le sujet

- Plusieurs modes délivrances du médicament sont observés à l'officine;
- L'existence de pratiques de délivrance des médicaments appartenant aux tableaux de substances vénéneuses, sans prescription médicale, dans les officines des pays en voie de développement;
- L'exercice personnel de la pharmacie d'officine n'est pas toujours appliqué dans les officines.

### Contribution de notre étude à la connaissance

- Trois modes de délivrance ont été observés dans des proportions plus ou moins égales, avec une légère prédominance de la délivrance sur demande du patient dans les officines;
- L'organisation de l'espace à l'officine ne prévoyait pas des zones de confidentialité pour le client dans la quasi-totalité des officines enquêtées;
- La présence en nombre important d'un personnel auxiliaire «sans qualification en santé» dans les officines; l'absence d'un ordonnancier dans la totalité des officines enquêtées de la ville de N'Djaména.

## Remerciements

Les auteurs remercient la direction générale de la pharmacie, du médicament et des laboratoires et les pharmaciens responsables des officines qui ont autorisé l'accès aux officines pour la réalisation de l'étude.
